# Supplementary material for: Structural and Functional Insights into the Evolution of SARS-CoV-2 KP.3.1.1 Spike Protein
Source: bioRxiv. 2024 Dec 10:2024.12.10.627775. Preprint. [Version 1] doi: 10.1101/2024.12.10.627775 (PMC11661143; doi:10.1101/2024.12.10.627775)
Supplement: Supplement 1 [file media-1.pdf]

Supplemental Information

Structural and Functional Insights into the Evolution of SARS-CoV-2 KP.3.1.1 Spike Protein

Ziqi Feng[1], Jiachen Huang[1], Sabyasachi Baboo[2], Jolene K. Diedrich[2], Sandhya Bangaru[1], James C. Paulson[2,3], John R. Yates III[2], Meng Yuan[1], Ian A. Wilson[1,4,*], Andrew B. Ward[1,5,*]

[1]Department of Integrative Structural and Computational Biology, The Scripps Research Institute, La Jolla, CA 92037, USA.

[2]Department of Molecular Medicine, The Scripps Research Institute, La Jolla, CA 92037, USA.

[3]Department of Immunology and Microbiology, The Scripps Research Institute, La Jolla, CA 92037, USA.

[4]The Skaggs Institute for Chemical Biology, The Scripps Research Institute, La Jolla, CA 92037, USA.

Correspondence: wilson@scripps.edu, andrew@scripps.edu

[5]Lead contact: andrew@scripps.edu

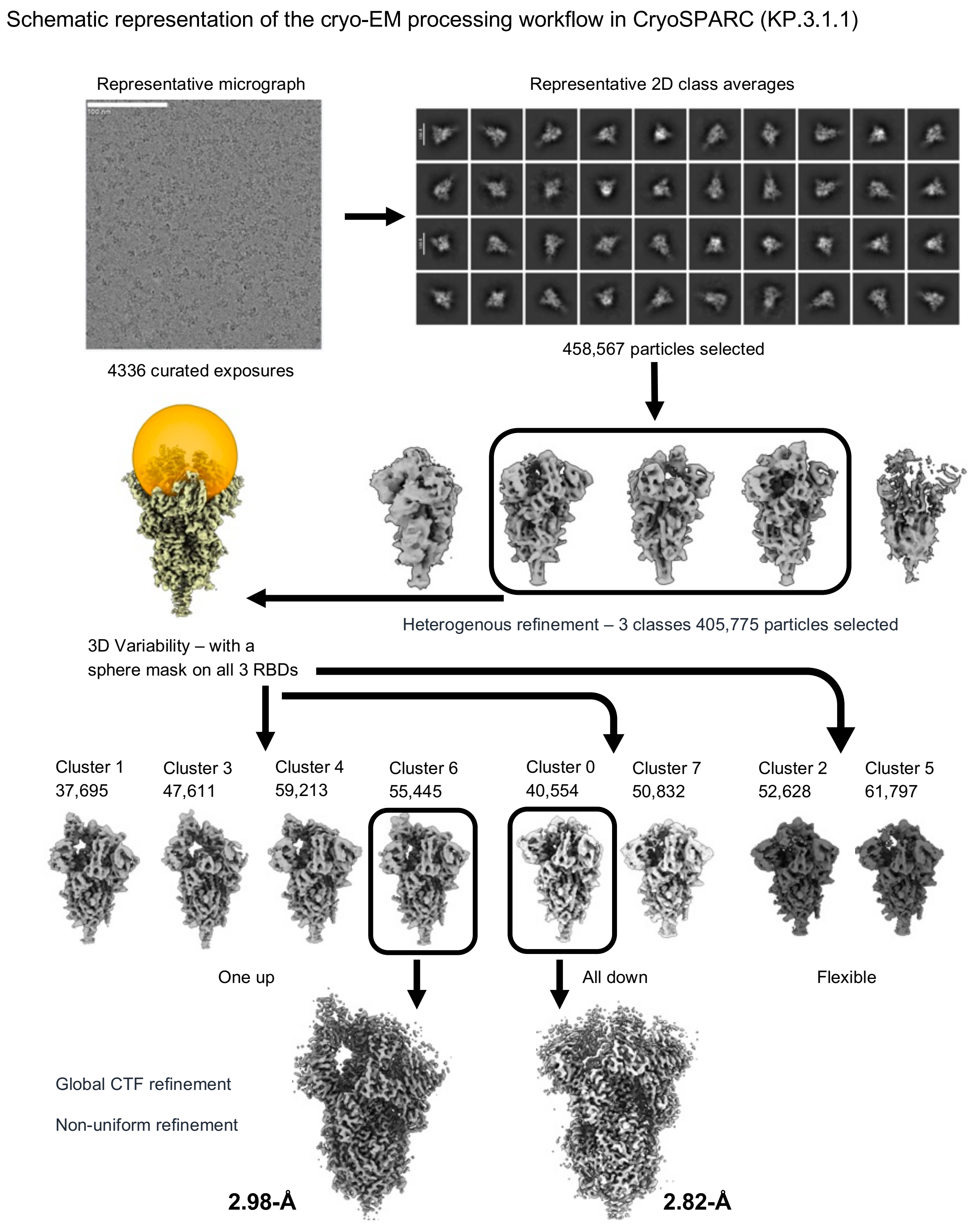

Figure S1. Schematic representation of the cryo-EM processing workflow for SARS-CoV-2 KP.3.1.1 apo spike. Workflow in cryoSPARC including initial processing steps (motion correction, CTF estimation, micrograph selection, particle picking, and selection based on 2D classification and heterogenous refinement) and 3D variability with a sphere mask on all three RBDs. All

particles were then separated into eight clusters and classified by their conformation. A representative map from each class underwent global CTF refinement and non-uniform refinement and was used for model building. This workflow has been applied to all datasets.

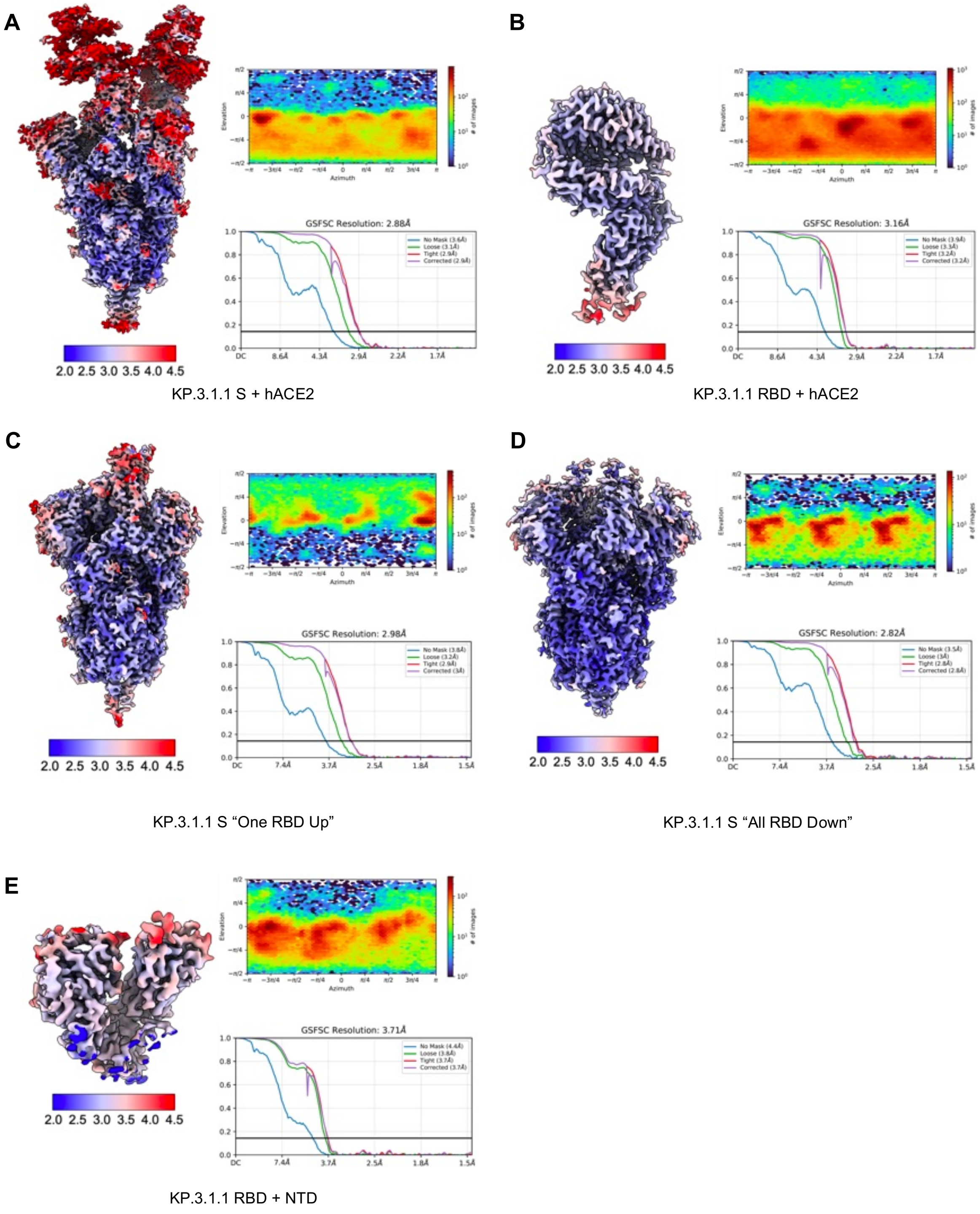
A
B
C
D
E
GSFSC Resolution: 2.88Å
GSFSC Resolution: 3.16Å
GSFSC Resolution: 2.98Å
GSFSC Resolution: 2.82Å
GSFSC Resolution: 3.71Å
2.0 2.5 3.0 3.5 4.0 4.5
Elevation
Azimuth
# of images
KP.3.1.1 S + hACE2
KP.3.1.1 RBD + hACE2
KP.3.1.1 S “One RBD Up”
KP.3.1.1 S “All RBD Down”
KP.3.1.1 RBD + NTD

F

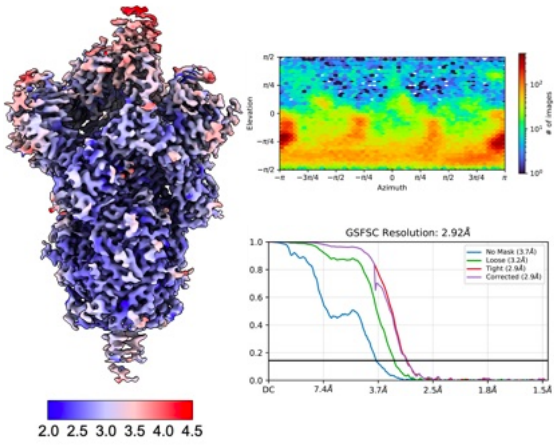

JN.1.11+S31Δ+Q493E S "One RBD Up"

G

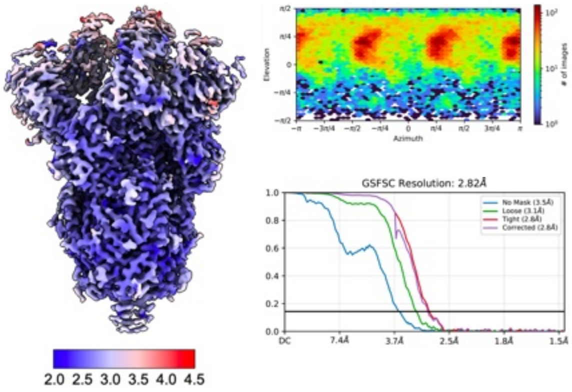

JN.1.11+S31Δ+Q493E S "All RBD Down"

H

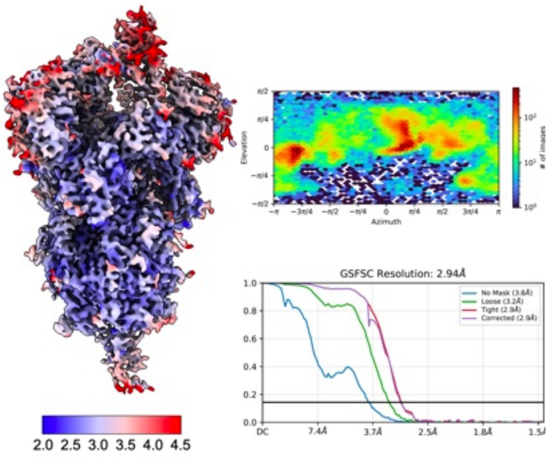

JN.1.11.1+S31Δ S "One RBD Up"

I

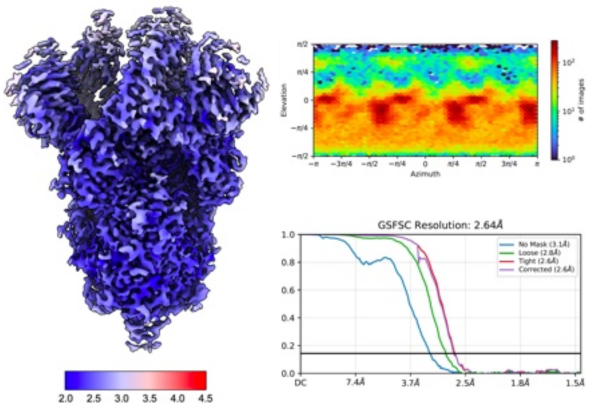

JN.1.11.1+S31 Δ S "All RBD Down"

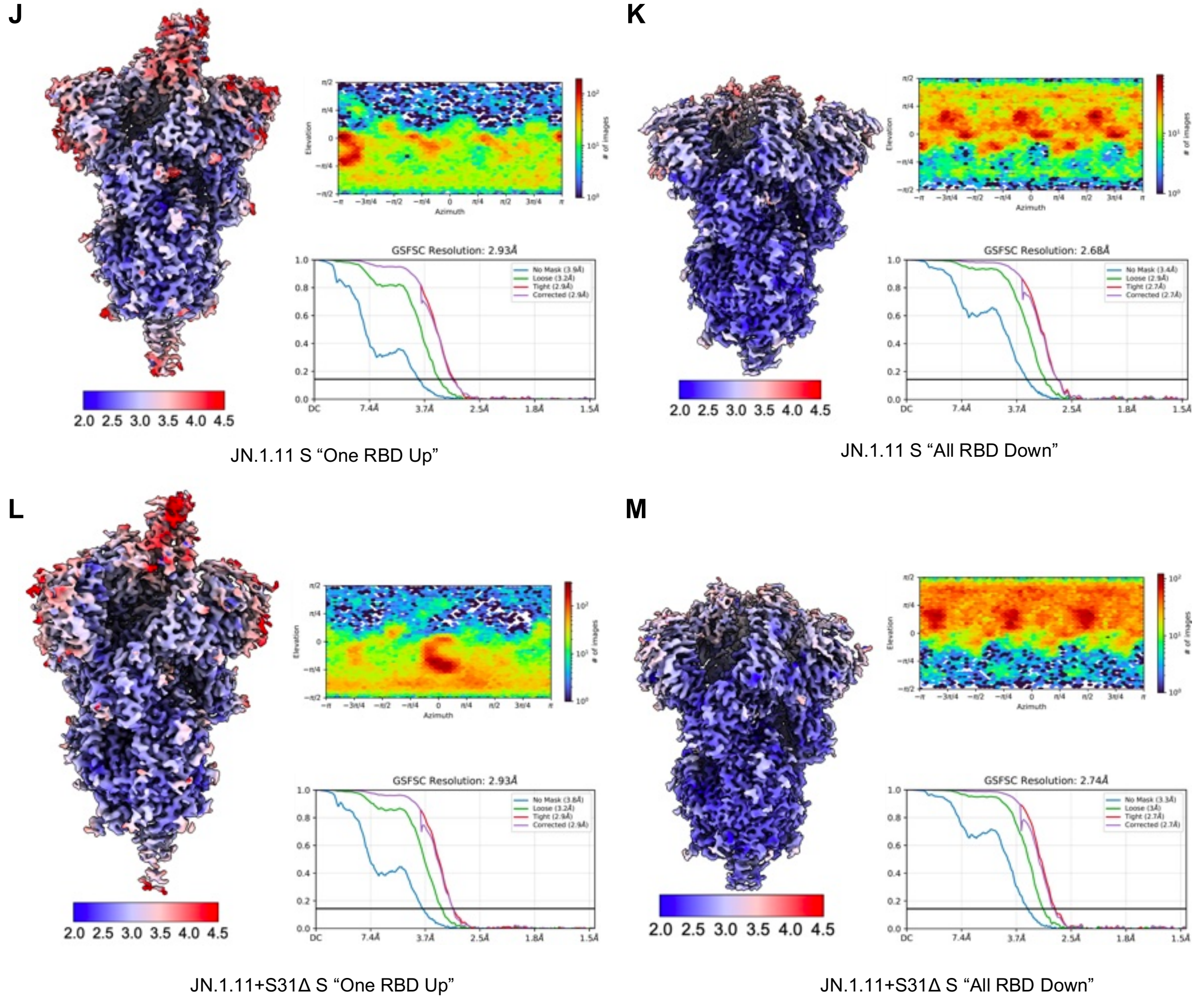

Figure S2. Angular distribution, corresponding FSC curves, and resolution distribution of the cryo-EM map. In the global and local resolution distribution of the cryo-EM maps, blue represents the high-resolution areas (2 Å) while red represents the low-resolution (4.5Å). **(A)** KP.3.1.1 S + hACE2. **(B)** KP.3.1.1 RBD + hACE2. **(C)** KP.3.1.1 S “One RBD Up”. **(D)** KP.3.1.1 S “All RBD Down”. **(E)** KP.3.1.1 RBD + NTD. **(F)** JN.1.11+S31Δ+Q493E S “One RBD Up”. **(G)** JN.1.11+S31Δ+Q493E S “All RBD Down”. **(H)** JN.1.11.1+S31Δ S “One RBD Up”. **(I)** JN.1.11.1+S31 Δ S “All RBD Down”. **(J)** JN.1.11 S “One RBD Up”. **(K)** JN.1.11 S “All RBD Down”. **(L)** JN.1.11+S31Δ S “One RBD Up”. **(M)** JN.1.11+S31Δ S “All RBD Down”.

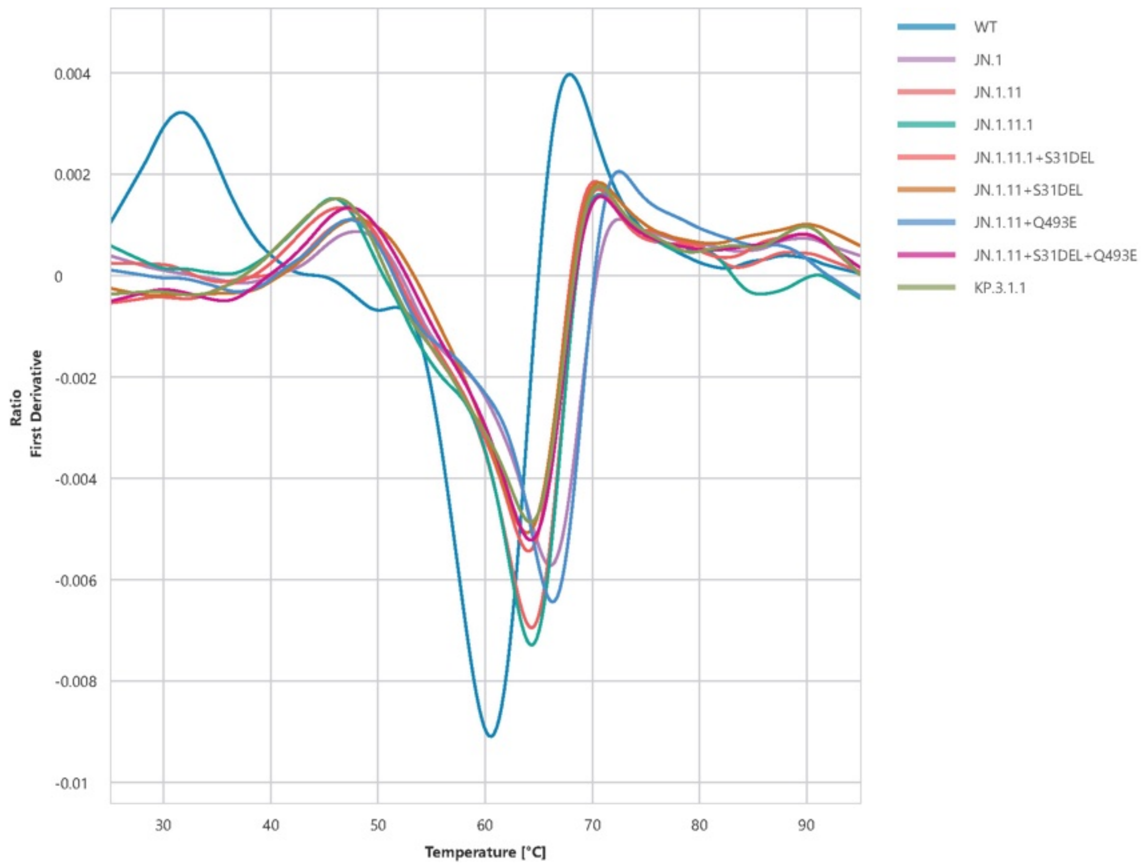

Sample	Tm (°C)
WT	31.65
JN.1	47.87
JN.1.11	47.59
JN.1.11.1	45.71
JN.1.11.1+S31DEL	46.46
JN.1.11+S31DEL	48.03
JN.1.11+Q493E	47.29
JN.1.11+S31DEL+Q493E	47.31
KP.3.1.1	46.01

Figure S3. Differential scanning fluorimetry analysis of spike variant thermostability.

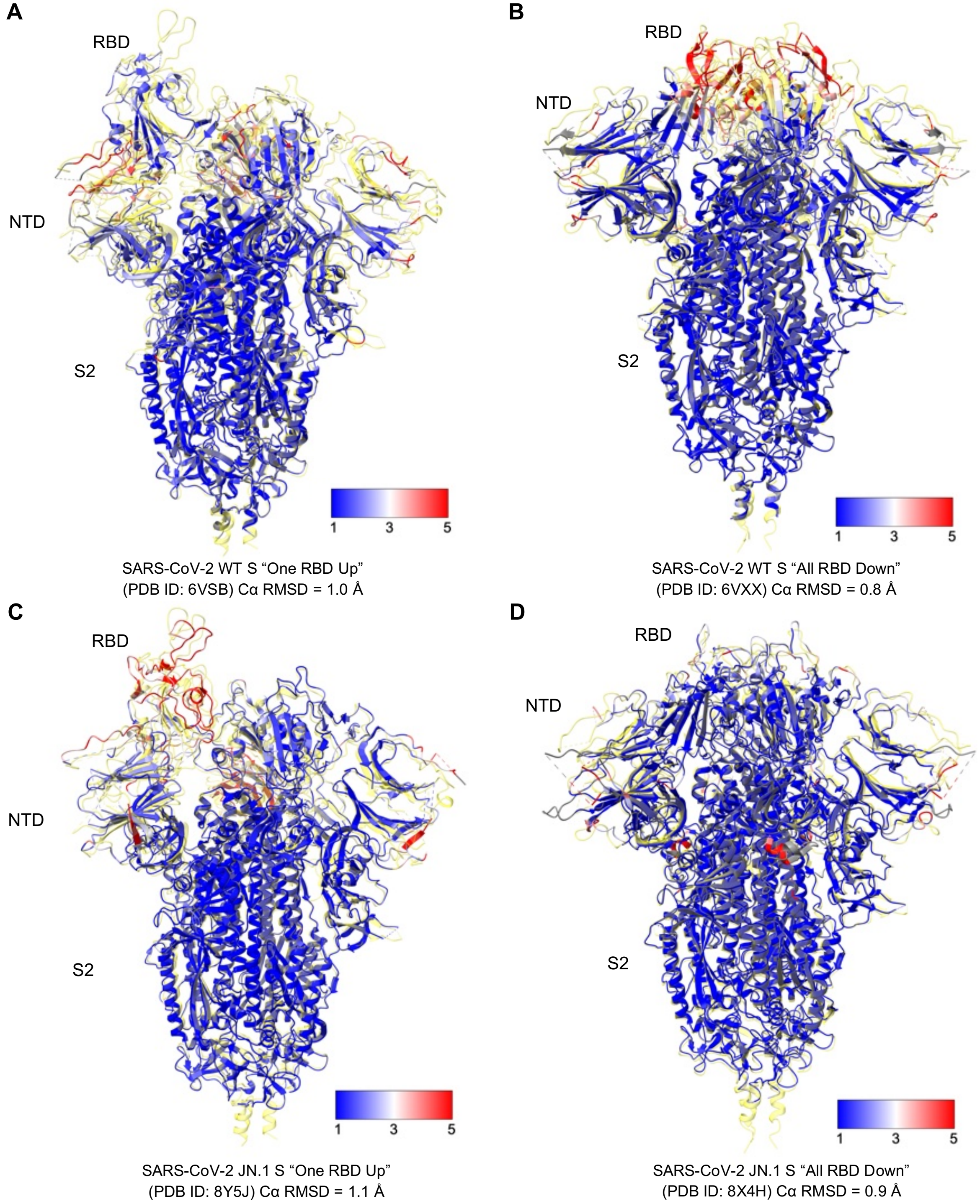
A
RBD
NTD
S2
1
3
5
SARS-CoV-2 WT S “One RBD Up”
(PDB ID: 6VSB) Cα RMSD = 1.0 Å
B
RBD
NTD
S2
1
3
5
SARS-CoV-2 WT S “All RBD Down”
(PDB ID: 6VXX) Cα RMSD = 0.8 Å
C
RBD
NTD
S2
1
3
5
SARS-CoV-2 JN.1 S “One RBD Up”
(PDB ID: 8Y5J) Cα RMSD = 1.1 Å
D
RBD
NTD
S2
1
3
5
SARS-CoV-2 JN.1 S “All RBD Down”
(PDB ID: 8X4H) Cα RMSD = 0.9 Å

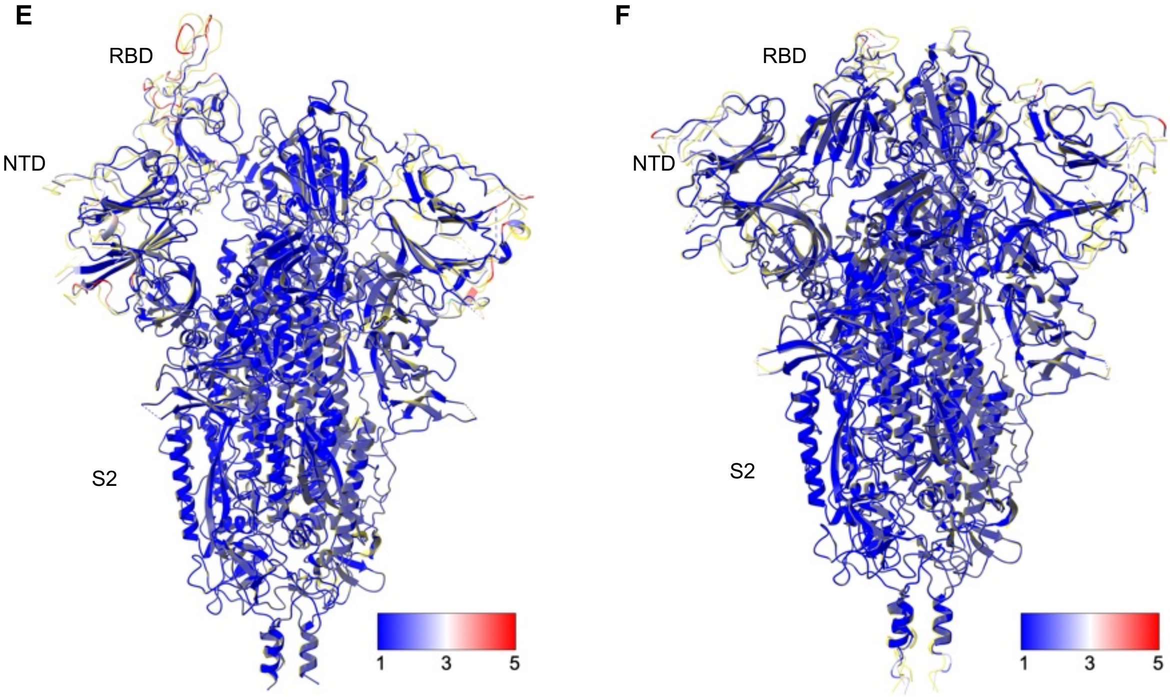

SARS-CoV-2 JN.1.11 S "One RBD Up"
(This study) Cα RMSD = 0.6 Å

SARS-CoV-2 JN.1.11 S "All RBD Down"
(This study) Cα RMSD = 0.5 Å

Figure S4. Structural superimposition of SARS-CoV-2 WT, JN.1, and JN.1.11 spike protein with KP.3.1.1 spike protein. KP.3.1.1 spike proteins are colored light yellow. Structural differences of SARS-CoV-2 WT, JN.1, and JN.1.11 spike are color-coded by their root mean square deviation (RMSD) (Å). Positions of RBD, NTD, and S2 are labeled. **(A, C, E)** Structural alignment of the KP.3.1.1 "One RBD Up" spike structure to SARS-CoV-2 WT (PDB ID: 6VSB), JN.1 (PDB ID: 8Y5J), and JN.1.11 (this study), respectively. **(B, D, F)** Structural alignment of the KP.3.1.1 "All RBD Down" spike structure to SARS-CoV-2 WT (PDB ID: 6VXX), JN.1 (PDB ID: 8X4H), and JN.1.11 (this study), respectively.

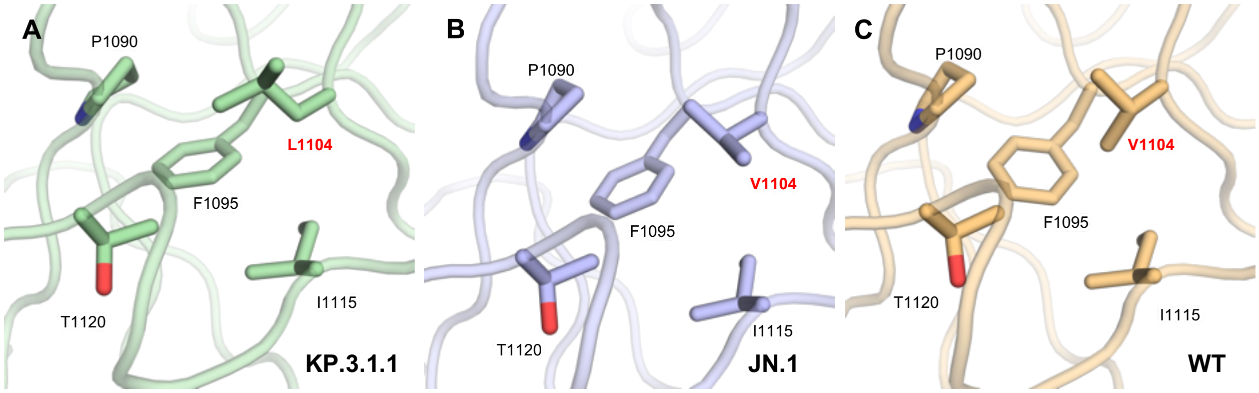

Figure S5. Zoomed-in view of V/L1104 in WT, JN.1, and KP.3.1.1 spikes. (A-C) L1104 or V1104 is surrounded by a hydrophobic pocket formed by P1090, F1095, I1115, and T1120 in KP3.1.1., JN.1, and WT spike proteins.

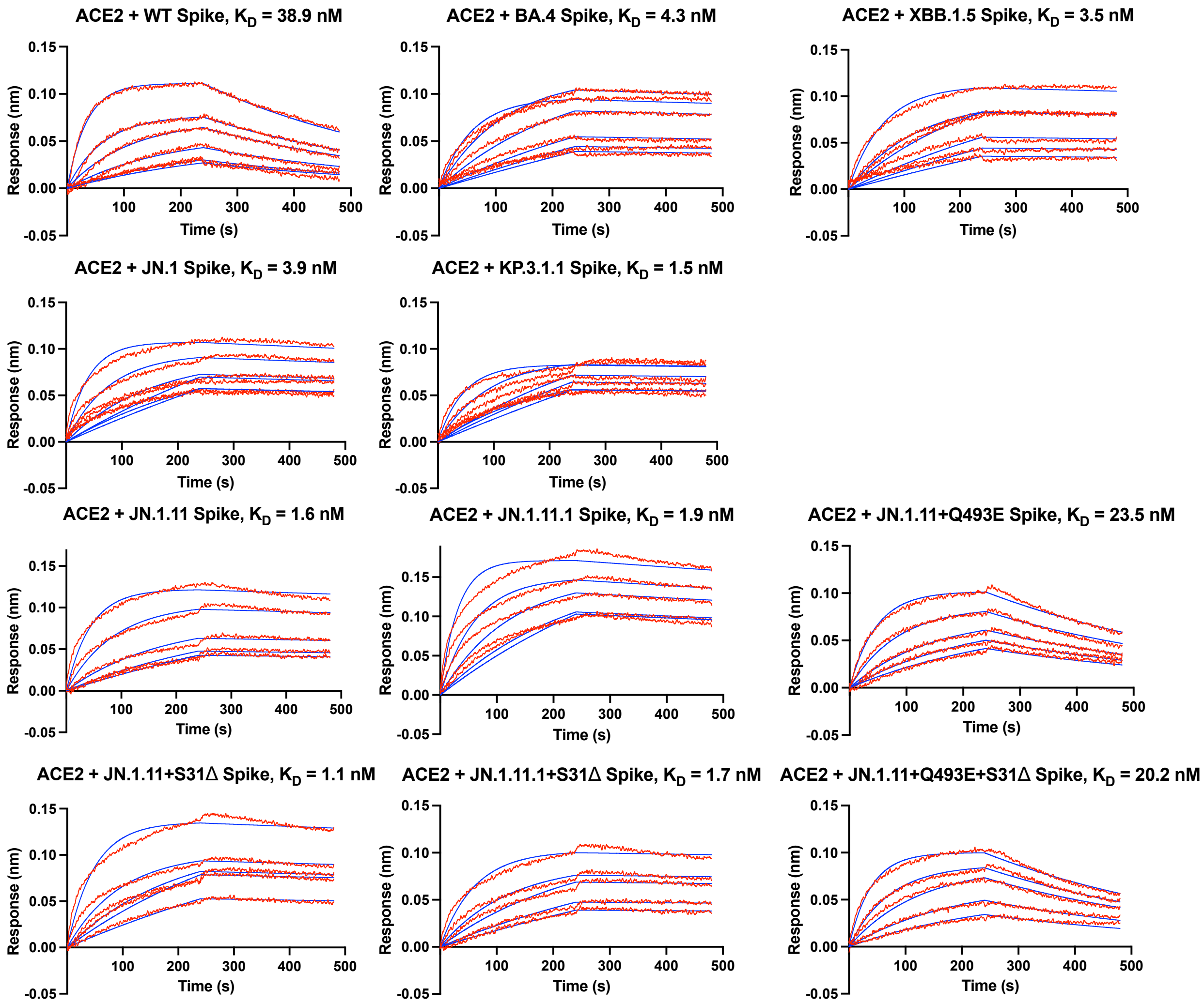

Figure S6. Sensorgrams for binding of various spikes to hACE2, related to Fig. 4F. Binding kinetics of different spike proteins against hACE2 were measured by biolayer interferometry (BLI). The Y-axis represents the response. Red lines represent the response curve, and blue lines represent a 1:1 binding model. Binding kinetics were measured for hACE2 concentrations (12.5, 25, 50, 100, 200, and 400 nM). Dissociation constant (K_D) is indicated.

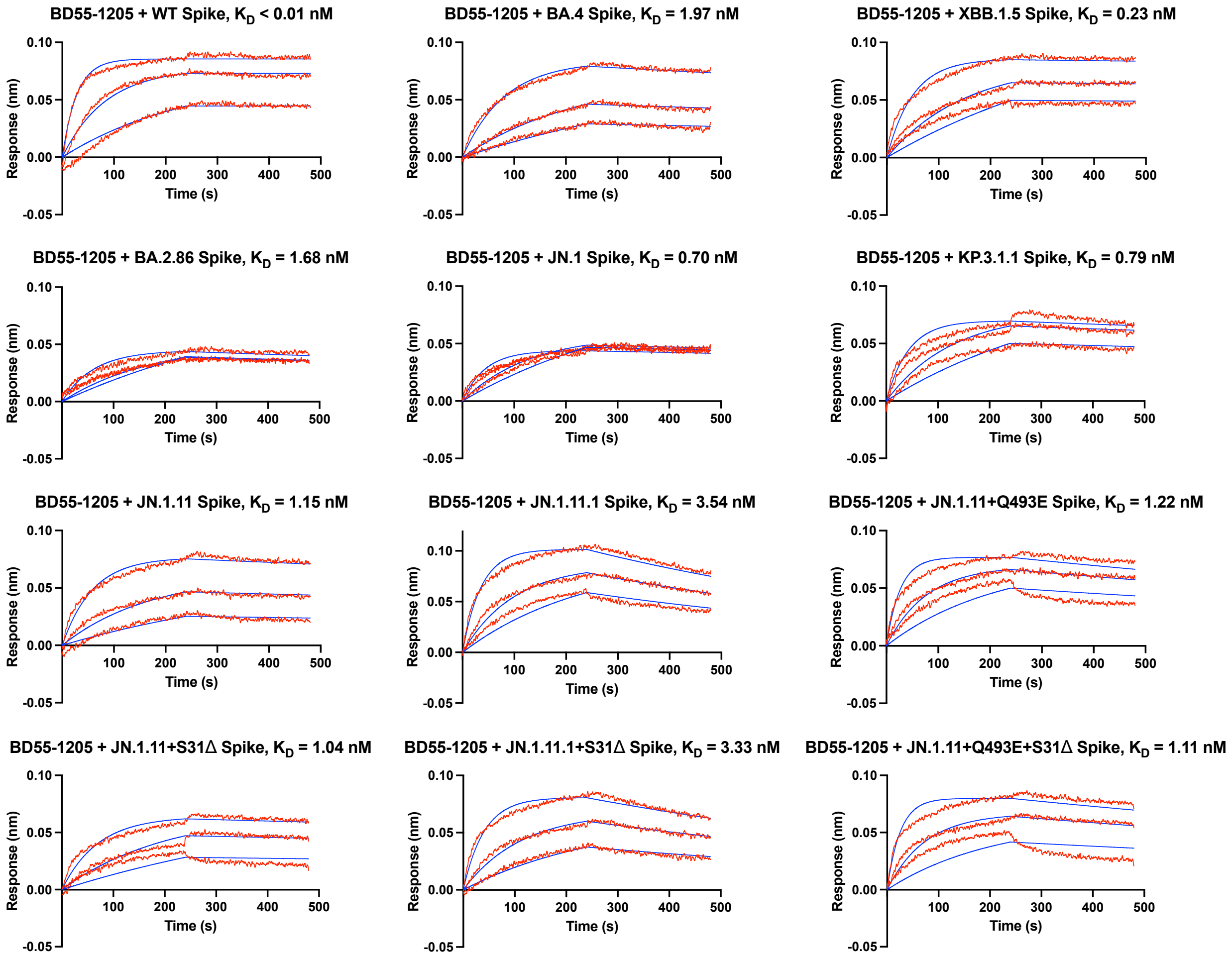

Figure S7. Sensorgrams for binding of various spikes to BD55-1205 Fab, related to Fig. 5F. Binding kinetics of different spike proteins against BD55-1205 Fab were measured by biolayer interferometry (BLI). The Y-axis represents the response. Red lines represent the response curve, and blue lines represent a 1:1 binding model. Binding kinetics were measured for Fab concentrations (8.33, 25, and 75 nM). Dissociation constant (K_D) is indicated.

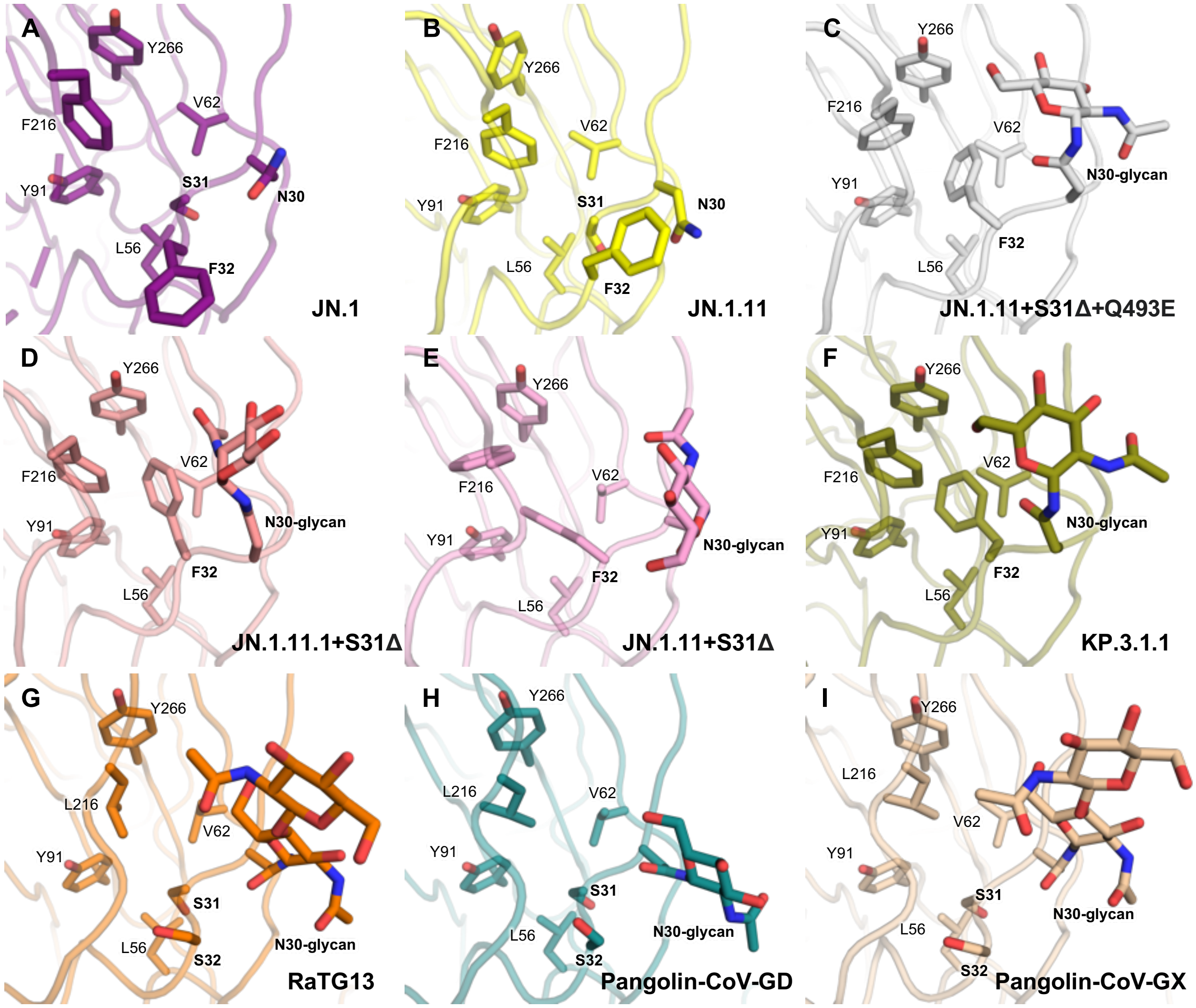

Figure S8. Zoomed-in view of N30 in various spikes. (A-I) Detailed side chain conformation including N30 with glycan, S31, F/S32, L56, V62, Y91, L/F216, and Y266 of JN.1 (purple), JN.1.11 (yellow), JN.1.11+S31Δ+Q493E (grey), JN.1.11.1+S31Δ (flesh), JN.1.11+S31Δ (pink), KP.3.1.1 (olive), RaTG13 (orange), Pangolin-CoV-GD (teal), and Pangolin-CoV-GX (wheat), respectively.

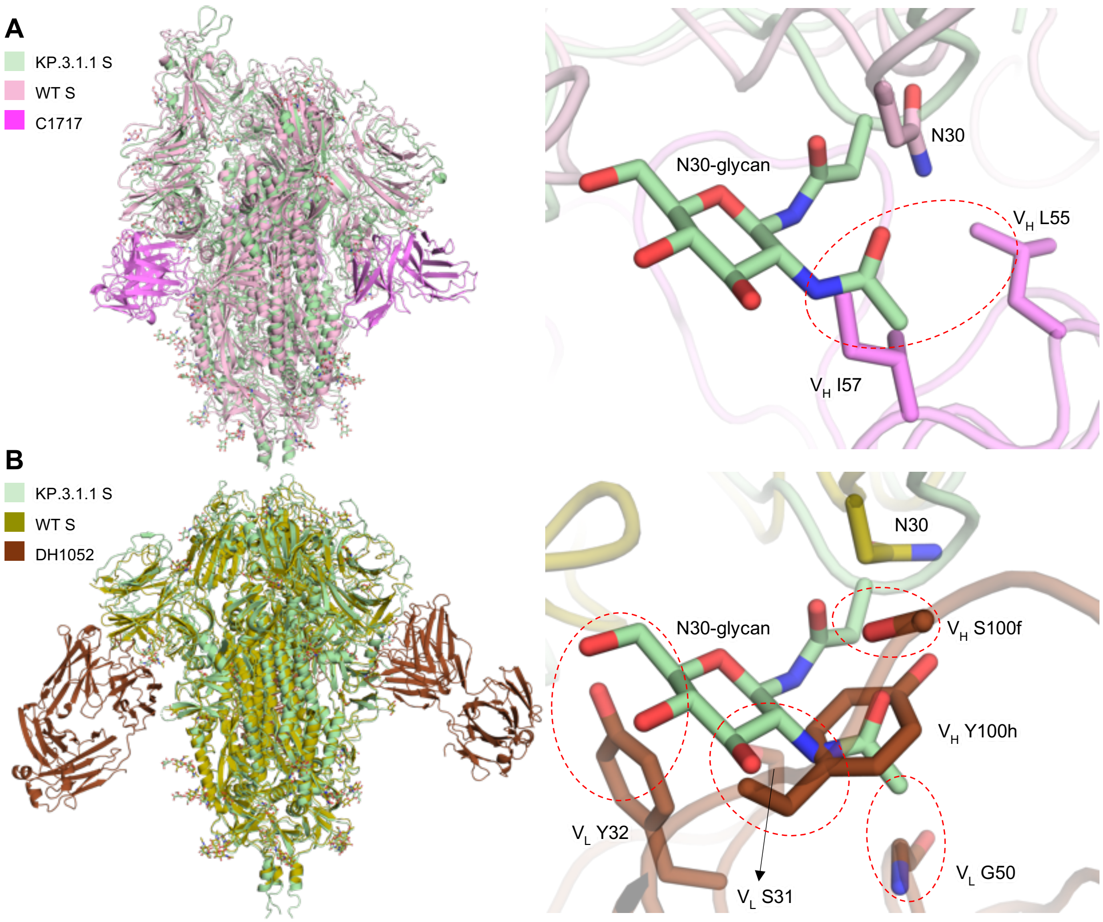

Figure S9. Structural alignment between KP.3.1.1 S and NTD site vi antibodies. (A) Left, superimposed structures of KP.3.1.1 S and WT S/C1717 complex. KP.3.1.1 S, WT S, and C1717 are colored in light green, pink, and magenta, respectively. Right, a zoomed-in view of the NTD shows that N30 glycan may clash with C1717 V_H L55 and V_H I57. The red dashed circle highlights the potential clash. **(B)** Right, superimposed structures of KP.3.1.1 S and WT S/DH1052 complex. KP.3.1.1 S, WT S, and DH1052 are colored in light green, olive, and brown, respectively. Right, a zoomed-in view of the NTD shows that N30 glycan may clash with DH1052 V_H S100f, Y100h and V_L S31, Y32, G50. Red dashed circles highlight the potential clashes.

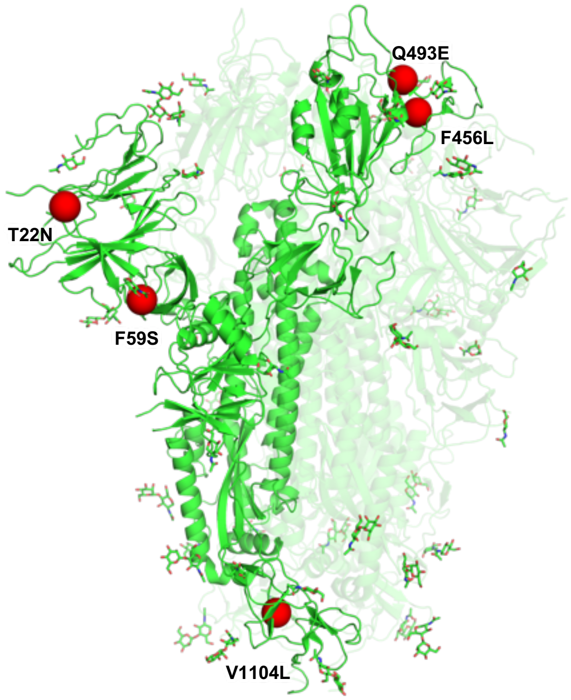

Figure S10. Mutations (red) on XEC in the context of JN.1 S structure (green) (PDB ID: 8X4H).

Table S1. Cryo-EM data collection, processing, and model building statistics.

Sample	KP.3.1.1 Spike HexaPro + hACE2		KP.3.1.1 Spike HexaPro			JN.1.11 S31del Q493E Spike HexaPro		JN.1.11 Spike HexaPro		JN.1.11 + S31del Spike HexaPro		JN.1.11.1+S31del Spike HexaPro	
**Map**	Local refine RBD+hACE2	Global refine S with two hACE2	Local refine RBD+NTD	One RBD Up	All RBD Down	One RBD Up	All RBD Down	One RBD Up	All RBD Down	One RBD Up	All RBD Down	One RBD Up	All RBD Down
**EMDB**	48146	48147	48148	48149	48150	48151	48152	48153	48155	48156	48157	48158	48159
**PDB**	9ELE	9ELF	9ELG	9ELH	9ELI	9ELJ	9ELK	9ELL	9ELM	9ELN	9ELO	9ELP	9ELQ
**Data collection & processing**													
**Microscope/ Detector**	Glacios/Falcon4												
**Voltage (kV)**	200												
**Magnification**	190,000												
**Recording mode**	Counting												
**Pixel size (Å)**	0.718												
**Total dose (e^-/Å^2)**	44.84												
**Defocus range (μm)**	-0.8 to -1.7												
**No. of movie micrographs**	4334		4336			4716		3680		4010		7672	
**No. of molecular projection images in map**	440,016	131,224	98,127	55,445	40,554	94,590	41,085	39,569	42460	63,034	59,178	53,372	112,912
**Symmetry**	C1	C1	C1	C1	C3	C1	C3	C1	C3	C1	C3	C1	C3
**Map pixel size (Å)**	0.718												
**Map resolution (FSC 0.143; Å)**	3.16	2.88	3.71	2.98	2.82	2.92	2.82	2.93	2.68	2.93	2.74	2.94	2.64
**Map sharpening B-factor (Å^2)**	-107.1	-66.1	-110.6	-58.9	-72.6	-65.5	-68.0	-48.7	-62.7	-58.2	-72.1	-53.3	-76.2
**Structure building and validation**													
**Model composition**													
**Non-hydrogen atoms**	6529	35225	4255	25512	25509	25558	25299	25567	25275	25446	25446	23543	25479
**Protein residues**	780	4334	510	3152	3153	3159	3123	3157	3117	3150	3150	2916	3150
**Ligands**	NAG:12/BMA:1	NAG:58/ BMA:6	NAG:11	NAG:52/ BMA:7	NAG:51/ BMA:6	NAG:50/ BMA:7	NAG:51/ BMA:6	NAG:52/ BMA:7	NAG:51/ BMA:6	NAG:49/ BMA:7	NAG:48/ BMA:6	NAG:47/ BMA:7	NAG:51/ BMA:6
**RMSD bond length (Å)/angles (°)**	0.007/ 1.20	0.007/ 1.23	0.008/ 1.28	0.007/ 1.16	0.017/ 1.19	0.006/ 1.09	0.007/ 1.16	0.007/ 1.13	0.009/ 1.41	0.012/ 1.37	0.008/ 1.29	0.008/ 1.21	0.008/ 1.33
**MolProbity score**	1.07	1.38	1.32	1.53	1.20	1.39	1.34	1.55	1.21	1.63	1.47	1.26	1.56
**Clash score**	2.83	6.92	2.76	5.32	2.18	3.53	4.15	5.83	4.15	6.02	5.75	2.69	7.59
**Ramachandran outliers/allowed/favored (%)**	0/ 1.03/ 98.97	0/ 1.94/ 98.06	0/ 3.82/ 96.18	0/ 3.64/ 96.36	0/ 3.32/ 96.68	0/ 3.60/ 96.37	0/ 2.73/ 97.27	0/ 3.57/ 96.43	0/ 2.05/ 97.95	0.03/ 4.23/ 95.74	0/ 2.90/ 97.10	0.03/ 3.26/ 96.71	0/ 2.80/ 97.20
**Rotamer outliers (%)**	0	0.13	0	0.04	0	0	0	0.14	0	0.25	0.11	0.16	0.11
**Cβ outliers (%)**	0	0.32	0.63	0	0.1	0	0	0	0	0.14	0.1	0.07	0.3
**d FSC model (0.5; Å)**	3.3	3.1	3.9	3.2	3.0	3.1	3.0	3.1	2.9	3.1	2.9	3.1	2.8
